# Community uptake of safe storage boxes to reduce self-poisoning from pesticides in rural Sri Lanka

**DOI:** 10.1186/1471-2458-7-13

**Published:** 2007-01-26

**Authors:** Flemming Konradsen, Ravi Pieris, Manjula Weerasinghe, Wim van der Hoek, Michael Eddleston, Andrew H Dawson

**Affiliations:** 1 Department of International Health, University of Copenhagen, Øster Farimagsgade 5, Building 16, P.O.Box 2099, 1014 Cph K, Denmark; 2 South Asian Clinical Toxicology Research Collaboration, Faculty of Medicine, University of Peradeniya, Sri Lanka; 3 Center for Tropical Medicine, Nuffield Department of Clinical Medicine, University of Oxford, UK; 4 Department of Clinical Medicine, University of Peradeniya, Peradeniya, Sri Lanka; 5 School of Population Health, University of Newcastle, Newcastle, Australia

## Abstract

**Background:**

Acute poisoning by agricultural pesticides is a well established global public health problem. Keeping pesticides under safe storage is now promoted as a potential way to reduce the number of severe poisoning cases. However, there have been no published studies documenting the feasibility of such an approach. Therefore, the objective of the study presented here was to determine community perceptions and use of in-house safe storage boxes for pesticides in rural Sri Lanka.

**Methods:**

Boxes with a lock, to be used for the in-house safe storage of pesticides, were distributed to 200 randomly selected farming households in two agricultural communities. A baseline survey determined pesticide storage practices and household characteristics prior to distribution. The selected households were encouraged to make use of the box at community meetings and during a single visit to each household one month after distribution. No further encouragement was offered. A follow-up survey assessed storage practices seven months into the project.

**Results:**

Following the distribution of the boxes the community identified a number of benefits including the protection of pesticide containers against exposure from the rain and sun and a reduced risk of theft. Data were analysed for 172 households that reported agricultural use of pesticides at follow-up. Of these, 141 (82%) kept pesticides in the house under lock against 3 (2%) at baseline. As expected, the distribution of boxes significantly reduced the number of households storing pesticides in the field, from 79 (46%) at baseline to 4 (2%) at follow-up. There was a significant increase in the number of households keeping pesticides safe from children between baseline (64%) and seven months after the distribution of boxes (89%). The same was true for adults although less pronounced with 51% at baseline and 66% at follow-up.

**Conclusion:**

The farming community appreciated the storage boxes and made storage of pesticides safer, especially for children. It seems that additional, intensive promotion is needed to ensure that pesticide boxes are locked. The introduction of in-house safe storage boxes resulted in a shift of storage into the farmer's home and away from the field and this may increase the domestic risk of impulsive self-poisoning episodes. This increased risk needs attention in future safe storage promotion projects.

## Background

Acute poisoning by agricultural pesticides is a well established public health problem across the developing world with an estimated 300,000 deaths globally every year [1]. Research over the past 10 years has shown that the great majority of deaths follow impulsive acts of self-harm where the ready availability of pesticides in the homes of rural communities plays a key role [2,3]. The WHO now estimates that pesticide ingestion is the most common method of suicide worldwide and has thus launched a global *Pesticides and Health Initiative *aimed at developing strategies to reduce the health impact of pesticides [4,5].

Keeping pesticides under safe storage is now promoted as a potential way to reduce the number of severe poisoning cases by organizations such as the WHO [5], the UN Food and Agricultural Organisation (FAO) [6], and the global federation representing pesticide manufacturers, CropLife [7].

However, there have been no published studies documenting how the introduction of such programs changes the location of storage, and the acceptability and actual use of such storage devices in low income countries. In particular, there is no evidence that promotion of safe pesticide storage reduces the number of severe poisoning cases.

Before initiating a large scale epidemiological study to test the effectiveness of safe storage, we needed to understand community acceptance, priorities, and preferences. We therefore set up a study in a rural area of Sri Lanka to determine community perceptions and use of in-house safe storage boxes for pesticides.

## Methods

### Study area

The study was carried out in an irrigated resettlement area of the North Central Province of Sri Lanka, focussing on two villages, labelled villages "S" and "R", located eight kilometres apart. Activities were initiated in February 2005. The two villages were selected from a list of nine villages because they had a high rate of deliberate pesticide poisoning cases, according to local hospital in-patient records for the previous year.

Approval for the collection of information from health facilities and the collection of follow-up for health outcomes following poisoning have been received from the Sri Lankan Medical Association Ethics Committee and from the relevant Ministry of Health Departments.

The two villages represented differences with respect to agricultural production and history of settlement. Village R had access to irrigation water for both cultivation seasons, *maha *(wet season generally from late November to early April) and *yala *(dry season – generally from late April to late October), for paddy or vegetable production. Its farmers purchased pesticides from outlets located in the centre of the settlement. Almost all of the 341 households registered in the village settled in the area in 1966 or thereafter. At the time of settlement, they had been allocated 2.5 acres of cultivation land and 0.5 acres of homegarden. At the start of this study, 152 of the households were registered as being involved in farming.

Village S had 298 households with 200 involved in farming. Most families had lived in the area for generations and were involved in irrigated paddy agriculture during the *maha *season. In the *yala *season, 20 to 25 of the households were able to cultivate vegetables while most other households farmed small horticultural or agro-forestry plots around the house or as part of a slash and burn cultivation systems. The diversity in the size of landholding was greater in village S than village R. Also, in village S a significant monetary income was generated from off-farm employment including employment in the garment industry or from family members working overseas. The farmers purchased pesticides from outlets located five kilometres away.

Pesticides were promoted in the two villages by sales representatives but most marketing activities took place at outlets or on large billboards displayed along the main roads. Neither government nor non-governmental organisations were active in raising the awareness of the hazards of pesticides or how to store pesticide safely. The general patterns of pesticide use and poisoning in the study area have been described [8,9].

### Study population

**Figure 1:**
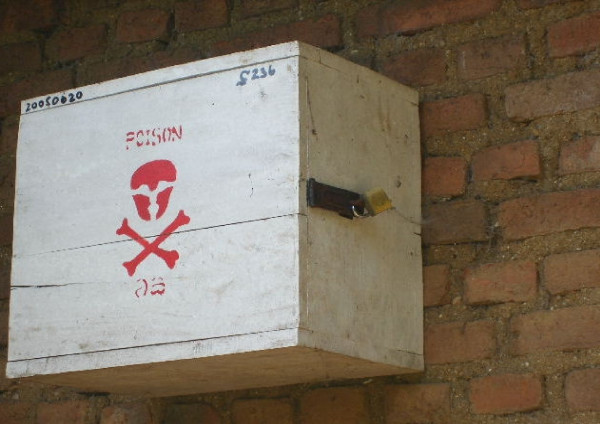
Wooden box with a pad lock for the safe storage of pesticides.

**Figure 2:**
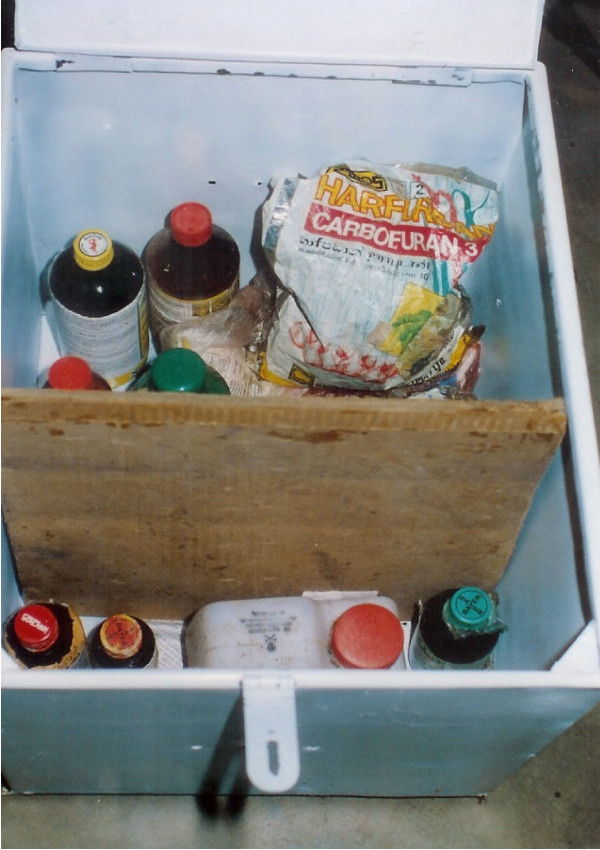
Pesticides stored in metal box for the safe storage of pesticides.

After selecting the two villages, the purpose of the study was presented to the community at a meeting in each village, organized by the head of the farmer organisation and the government representative in the village, and a discussion on pesticide use and storage of pesticides was initiated. After the aims of the project had been presented the members of the community present at both village meetings agreed to take part when asked for an expression of interest by the project staff. Based on farmers' preferences two different boxes were developed but only one box was made available to each household. The metal box was 45 cm × 30 cm × 37 cm (approximately 18 inches × 12 inches × 14 inches) with a local cost of production of Rs. 980 (USD 9.5). The wooden box was 42 × 35 × 35 cm (16 inches × 14 inches × 14 inches) at a production cost of Rs. 1400 (USD 13.5). For both type of boxes the cost include a padlock (see Figure 1 and 2).

In April 2005, a second community meeting was arranged where 100 households in each village were selected from the village list of registered farming households. The selection was based on a lottery where a representative from the farmer organisation and the head monk drew numbers. At a visit to each of the selected households the purpose of the project was outlined and the head of household was asked for consent before a final selection of study households was made. Two farming households, in village S, refused to take part and were replaced by two new households from the same village.

### Focus group discussions and transect walks

Prior to the initiation of a baseline survey a total of twelve focus group discussions were held separately with male farmers and female representatives, approximately ten in each group, to discuss the perceived advantages and disadvantages of pesticide storage boxes, general issues of pesticide use and storage, and possible health impacts of pesticides.

The research team during the baseline survey conducted transect walks throughout the village and agricultural fields allowing for opportunities to talk to community members and to identify patterns of pesticide application and storage.

### Baseline survey

Immediately before box distribution, the selected households were interviewed using a questionnaire in the local language to obtain information on household composition, educational level, socio-economic status, agricultural practices, pesticide use and storage, and past history of pesticide poisoning in the household. All households practised Buddhism; religion was, therefore, not used in the analysis. Socio-economic status was defined in terms of assets and wealth rather than income. In a scoring system, one point was assigned to each of the following 9 items: ownership of a television, refrigerator, tractor, water pump, complete house construction, brick/plastered walls, tiled/asbestos roof; ownership of land, and cultivation of at least 2.5 acres of land. Households above the median were classified as having a high socio-economic status while those below were classified as having a low socio-economic status. Household educational level was based on the household member with the highest number of years at school. Households with a member who had completed secondary school (grade 10) were classified as having a 'high' educational level, the others as having a 'low' educational level.

Information on pesticide storage was based on direct observations by the interviewer who recorded whether pesticides were kept in the home or in a shed close to the home and whether or not they were kept under lock. Not all agricultural fields were inspected and information on storage of pesticides in the field was obtained from the household members. The baseline survey was planned to coincide with a period of high application of pesticides during April and May 2005.

During the baseline survey the head of the household and other family members present in the house were instructed on the use of the box. The head of household was also encouraged to identify a person within the household to carry the key to the padlock or alternatively two people, each carrying a key for different padlocks on the same box.

One to three weeks after the baseline survey, the households were provided with the safe storage box of their choice, free of charge, along with a heavy padlock and keys. At the point of distributing boxes it was made clear to the members of the household that they could opt out of the project at any time.

### Follow-up surveys

One month after box distribution, the communities' response to the installed boxes was assessed and the research team again emphasised the importance of storing all pesticides in the box and keeping it locked at all times. No effort was subsequently made to encourage use of the boxes and keeping them locked.

In December 2005 and January 2006, seven months after the distribution of boxes, a second follow-up survey was done coinciding with the maximum use of pesticides for paddy cultivation. Five households could not be followed-up as houses were closed during the survey.

In the remaining 195 households, the boxes were inspected and the households asked about pesticide applications during the past and present season. Twenty-three households reported that they had not used pesticides and were excluded from further analysis.

### Data analysis

Data were analysed for the 172 households that could be followed-up seven months after the baseline survey and that reported agricultural use of pesticides at that time. SPSS 10.01.1 was used for data analysis.

### Field research staff

Two male university graduates (RP and MW) conducted the field work. They jointly did 25 household visits at baseline and at follow-up surveys to ensure similar recording of data and reduce inter-observer variation. After the initial 25 household visits the two field researchers compared data sheets on observations, response to questions and the assessments made on storage of pesticides. All other household surveys were completed by a solo field researcher.

## Results

### Baseline survey

Ninety-four percent of households had cultivated paddy in the previous *maha *season and used herbicides for land preparation and after sowing. Vegetables were cultivated by 53% of households, generally on smaller plots, for which most used insecticides. In *yala *only 13% of households had cultivated paddy and 30% had cultivated vegetables due to lack of water for cultivation.

Table 1 shows baseline data for the use of pesticides by the 172 households that were available for follow-up. In line with the reduced agricultural activities in the *yala *season, pesticide use in *yala *was much lower than in *maha*. In *maha *there was heavy use of organophosphorus insecticides as well as herbicides.

**Table 1:** Baseline self reported pesticide use by 172 households in last *maha *and *yala *agricultural season.

	***maha***		***yala***	
	**No. (%) of households**	**No. of containers used**	**No. (%) of households**	**No. of containers used**

OP class Ib^1^	17 (10%)	28	3 (2%)	4
OP class II^2^	108 (63%)	231	27 (16%)	42
Carbamate class Ib^1^	26 (15%)	50	7 (4%)	25
Carbamate class II^2^	22 (13%)	39	1 (1%)	1
Paraquat^2^	57 (33%)	100	3 (2%)	3
Other herbicides	106 (62%)	348	12 (7%)	21
All other pesticides	46 (27%)	168	17 (10%)	27

^1 ^Classified as 'highly hazardous' [10]

^2 ^Classified as 'moderately hazardous' [10]

The two villages did not differ with respect to household size, presence of children or young adults, educational level, socioeconomic status, and paddy cultivation during maha (p ≤ 0.05 with χ^2 ^test, data not shown). Vegetable cultivation was more common in village R than in S (63% and 43% respectively, χ^2 ^= 6.74, p = 0.007) but this did not result in differences in quantity of pesticides used between the two villages (χ^2 ^= 0.37, p = 0.324).

The field research staff observed that 56 households (33%) kept pesticides in the house and 37 (22%) kept pesticides in a separate shed outside the main house but within the homestead (Table 2). In the remaining 79 houses (46%) no pesticides were seen and these households reported currently keeping their pesticides in the field located from less than hundred meters up to two kilometres from the house.

**Table 2:** Storage of pesticides among 172 households at baseline and seven months after distribution of pesticide safe storage boxes

**Storage**	**Baseline**	Follow-up survey*	**χ**^**2**^	***p***
Pesticides openly in house^1^	53 (31%)	27 (16%)	11.0	<0.001
Pesticides in house, under lock	3 (2%)	141 (82%)	227.5	<0.001
Pesticides in unlocked outhouse	34 (20%)	0	37.7	<0.001
Pesticides in locked outhouse	3 (2%)	0	3.0	0.124
Pesticides in field	79 (46%)	4 (2%)	89.3	<0.001

Total	172 (100%)	172 (100%)		

^1 ^includes pesticides in unlocked box

Households that had experienced a pesticide poisoning incident in the last 3 months (1 household out of 165 responding households), or pesticide poisoning requiring hospital admission (30 households out of 142 responding households) or death at any time (12 households out of 158 responding households), did not differ in pesticide storage practices at baseline from those that had not experienced such an adverse event (data not shown).

### Follow-up survey

Out of the 172 households using pesticides, 170 (99%) still had the box while two had given it away to other family members to store pesticides. In 148 (86%) households, pesticides were stored in the box at the time of observation and in 141 (82%) the box was found to be locked (Table 2). Of the 27 households that still had pesticides openly in the house, five households had lost the key and the remaining 22 households considered it impractical to keep pesticides in a locked box when the pesticides were used frequently, or simply did not consider locking the box to be important.

### Safe storage

As no conventional definition has been established in the literature for safe storage of pesticides it was necessary for this study to come up with a definition that could be used in the context of in-house storage devices during both baseline and follow-up. This definition included the collection of objectively verifiable information (presence of a locked box and presence of pesticides in the house or homestead) as well as a qualitative assessment made by the research assistants during the baseline and the follow up visit to the households (accessibility of pesticides by children and adults and practices of keeping the key hidden).

A household was recorded as having safe storage if there were no containers in the house or in the homestead that would be accessible with minor effort, e.g. standing on a chair in the case of children or climbing a ladder in the case of adults. Pesticides in a locked box were considered safe from children but safe from adults if only one adult member of the household knew the location of the key and kept it hidden from others or if two different adult members of the household held keys to separate locks of the same box. Pesticides stored in the field were considered safe to both children and adults.

Based upon the definition above it was found that there was a significant increase in the number of households keeping pesticides safe from children between baseline (64%) and seven months after the distribution of boxes (89%). The same was true for adults although less pronounced with 51% at baseline and 66% at follow-up (Table 3).

**Table 3:** Number and percentage of 172 households that stored pesticides safe from children and adults at baseline and seven months after distribution of pesticide safe storage boxes

	**Baseline**	**Follow-up**	**χ**^**2**^	***p***
**Children**				
Safe	110 (64%)	153 (89%)		
Not safe	62 (36%)	19 (11%)	29.86	<0.001
**Adults**				
Safe	87 (51%)	113 (66%)		
Not safe	85 (49%)	59 (34%)	8.07	0.004

When comparing households that kept pesticides safe from adults with those that had unsafe storage practices, no differences were found between the two villages (χ^2 ^= 0.34; *p *= 0.337) and in amount of pesticides used (χ^2 ^= 0.23; *p *= 0.374), socioeconomic status χ^2 ^= 0.00; *p *= 0.560) and educational level (χ^2 ^= 0.73; *p *= 0.475).

### Acute poisoning cases during the study period

During the study period, four severe acute poisoning cases where recorded from the follow up survey, three in village R and one in village S. All involved intentional self-poisoning. Two cases were from households that were provided with a box, including one person who died, and two from households without a box, in which one died. In one of the households provided with a box, a young woman succeeded in forcing open the metal box, drank the contents of a pesticide container, and died shortly after. In another case, a drunken elderly male farmer tried to force open a metal box when he could not find the key. He was not able to open it but instead bought pesticides from a shop and self-poisoned. He survived following hospitalization.

### Perceptions of farming households on the importance of safe storage and box design

Farming households considered safe storage to be an important issue and many families not selected for the study asked for a box from the project. Community members and local craftsmen were active participants in discussions about design options. The selection of locks was a particular problem since locally available locks were either too expensive or of poor quality.

Farmers reported that the locked boxes would be beneficial since they should reduce the risk of theft, bottles would not be misplaced easily, and the pesticides could be kept for longer since reduced sun and rain exposure would result in less damage to contents and label. They emphasised the importance of the box being large enough to store all pesticides and household chemicals and strong enough to stop people breaking into it.

During initial discussions and the first follow-up survey, community members were forthcoming in relating the risk of household pesticide storage to poisonings among children. However, as the household interviews progressed and a better rapport established, the importance of safe storage for preventing self-harm amongst vulnerable members of the community, in particular the mentally ill, emotionally distressed, or drunk was often raised. However, many respondents thought that it would be very difficult to prevent the use of pesticides for self-harm – as they were widely available outside the house – and prevent self-harm in general among adults.

During the household visits the poorer households emphasized the box's importance for reducing self-harm whereas richer households considered the box to be less important since they did not perceive themselves as vulnerable to self-harm. However, self-poisoning was well recognised by all members of the community and considered a problem.

The possibility of developing boxes for in-field storage, such as concrete containers buried in the ground, was brought up by some farmers as being more convenient and even safer than keeping pesticides at home. However, others argued that this would make the pesticides vulnerable to theft and make it possible for people to get access to them unnoticed, possibly increasing the likelihood of self-poisoning.

## Discussion

In general, the community appreciated the boxes and identifying a number of benefits such as improved protection of the pesticide containers against environmental factors and a reduced risk of theft. In addition, the provision of boxes in the households significantly improved storage safety, particularly for children. Their main health benefits according to the households were prevention of unintentional poisoning by small children and intentional poisonings by older children. However, if this is the case, since both unintentional and intentional paediatric poisoning make up only a small proportion of overall poisoning episodes, the impact of the improved storage practices on the total number of poisoning episodes may be limited [1].

The results presented here obviously depend on the definition and approach used to assess safe storage of pesticides, for which no common ground has yet been established in the literature. One key factor is how the storage of pesticides in the field is viewed. In this study, it was decided to regard pesticides stored in the field as safe; this provides a conservative estimate of benefit from the intervention. This assumption is supported by ongoing research in the same province where this study took place. Based on information collected from patients admitted to Polonnaruwa hospital in the North Central Province of Sri Lanka over a 18 months period, it was found that only 11% of the pesticide self-poisoning cases had accessed field-stored pesticides (ACM Fahim, unpublished observations). However, the implications of storage in the field will be site specific and the risk will have to be assessed for each location. Also, in future studies the distance to the field storage site and the conditions under which the pesticides are maintained in the field will have to be assessed in greater detail.

The household level management of the key to the locked box was part of the overall assessment done by the field research team to assess safe storage but did involve some difficulties. For example, it was difficult to assess if the children in a house or the partner to the person keeping the key truly did not know the location of the key or did not have access to the key. But in this pilot project the importance of keeping the box locked and the key hidden from the other family members was clearly advocated to the household members individually and at community meetings. However, after seven months, still only 66% used a locked box and maintained the pesticides safe from both adults and children. Maintaining even this level of compliance in future efforts is likely to require continuous promotional activity.

Following the distribution of boxes, there was a significant change in storage of pesticides from the field to the home. Consequently, a very high level of community compliance with the principles of safe storage in the house will be needed to not increase the risk of impulsive acts of self-harm. It is also possible that promotion of in-house pesticide storage may encourage farmers to keep left-over pesticides between applications and between seasons, increasing year round availability in the domestic environment. This finding calls for in-field storage devices that are difficult to break. A number of different prototypes are now being piloted in Sri Lanka. In future feasibility and impact studies the piloting of in-field storage devices should be considered.

## Conclusion

Provision of safe storage boxes had high community acceptance and utilisation in the short term but was associated with a significant change in the pattern of storage. They produced a modest improvement in safer storage for adults which suggest that alternate locking methods or more intensive promotion is needed to ensure that pesticide boxes are locked in a sustainable manner. If this is not successful, the shift to storage in the farmer's home rather than in the field, may even increase the domestic risk from impulsive self-poisoning episodes. Longer term follow-up studies are required on acceptability and utilisation before extensive deployment of in-house safe storage boxes.

## Competing interests

The author(s) declare that they have no competing interests.

## Authors' contributions

FK, RP and MW were involved in designing the study and the format for data collection. RP and MW were responsible for field data collection and data entry. FK and WvdH were responsible for data analysis and produced the first draft version of the paper. ME and AHD contributed with significant input to the revisions of the manuscript and provided significant intellectual content through out the project. All authors approved and agreed to the final manuscript.

## Pre-publication history

The pre-publication history for this paper can be accessed here:



## References

[B1] Gunnell D, Eddleston M (2003). Suicide by intentional ingestion of pesticides: A continuing tragedy in developing countries. International Journal of Epidemiology.

[B2] Eddleston M, Phillips MR (2004). Self poisoning with pesticides. British Medical Journal.

[B3] Konradsen F, van der Hoek W, Peiris P (2006). Reaching for the bottle of pesticide – a cry for help. Self-inflicted poisonings in Sri Lanka. Social Science and Medicine.

[B4] Bertolote JM, Fleischmann A, Butchart A, Besbelli N (2006). Suicide, suicide attempts and pesticides: A major hidden public health problem. Bulleting of the World Health Organisation.

[B5] WHO The Impact of Pesticides on Health: Preventing Intentional and Unintentional Deaths from Pesticide Poisoning. http://www.who.int/mental_health/prevention/suicide/en/PesticidesHealth2.pdf.

[B6] FAO (2002). International Code of Conduct on the Distribution and Use of Pesticides.

[B7] CropLife International (1998). Guidelines for the safe and effective use of crop protection products.

[B8] Van der Hoek W, Konradsen F, Atukorala K, Wanigadewa T (1998). Pesticide poisoning: A major health problem in Sri Lanka. Social Science and Medicine.

[B9] Eddleston M, Sheriff MH, Hawton K (1998). Deliberate self harm in Sri Lanka: An overlooked tragedy in the developing world. British Medical Journal.

[B10] WHO (2005). The WHO recommended classification of pesticides by hazard and guidelines to classification 2004. Geneva: WHO.

